# Geniposide Alleviates Glucocorticoid-Induced Inhibition of Osteogenic Differentiation in MC3T3-E1 Cells by ERK Pathway

**DOI:** 10.3389/fphar.2019.00411

**Published:** 2019-04-18

**Authors:** Baocheng Xie, Jiahuan Wu, Yongmei Li, Xuejun Wu, Zhanwei Zeng, Chenhui Zhou, Daohua Xu, Longhuo Wu

**Affiliations:** ^1^Guangdong Key Laboratory for Research and Development of Natural Drugs, The Public Service Platform of South China Sea for R&D Marine Biomedicine Resources, Marine Biomedical Research Institute, Guangdong Medical University, Zhanjiang, China; ^2^Department of Pharmacology, Institute of Traditional Chinese Medicine and New Pharmacy Development, Guangdong Medical University, Dongguan, China; ^3^School of Nursing, Guangdong Medical University, Dongguan, China; ^4^College of Pharmacy, Gannan Medical University, Ganzhou, China

**Keywords:** geniposide, osteogenic differentiation, glucocorticoid, osteoporosis, ERK pathway, GLP-1 receptor

## Abstract

Glucocorticoid (GC) therapy is the leading cause of secondary osteoporosis and the therapeutic and preventative drugs for GC-induced osteoporosis are limited. In this study, we investigated the protective effects of geniposide on dexamethasone (DEX)-induced osteogenic inhibition in MC3T3-E1 cells. The results showed that there was no obvious toxicity on MC3T3-E1 cells when geniposide was used at the doses ranging from 1 to 75 μM. In DEX-treated MC3T3-E1 cells, geniposide promoted the alkaline phosphatase (ALP) activity and the mineralization. In addition, geniposide also significantly increased the mRNA and protein expression of osteopontin (OPN), Runt-related transcription factor 2 (Runx2), and Osterix (Osx) in DEX-treated MC3T3-E1 cells. Furthermore, geniposide activated ERK pathway in DEX-treated MC3T3-E1 cells. The ERK activation inhibitor U0126 and glucagon-like peptide-1 (GLP-1) receptor antagonist exendin 9-39 abolished the geniposide-induced activation of ERK and inhibited the protective effect of geniposide. Taken together, our study revealed that geniposide alleviated GC-induced osteogenic suppression in MC3T3-E1 cells. The effect of geniposide was at least partially associated with activating ERK signaling pathway via GLP-1 receptor. Geniposide might be a potential therapeutic agent for GC-induced osteoporosis.

## Introduction

Osteoporosis is a common bone disease characterized by a low bone mineral density and the deterioration of bone microarchitecture, leading to increased risk of fracture (Kanis et al., 2009). Glucocorticoid (GC) administration is the leading cause of secondary osteoporosis and 30–50% of patients with chronic use of GCs have the high risk of fractures (Buehring et al., 2013; Compston, 2018). However, GCs are widely used for treating inflammatory and autoimmune disorders, such as rheumatoid arthritis and asthma (van der Goes et al., 2014; Guler-Yuksel et al., 2018). Therefore, it is imperative to develop drugs to treat GC-induced osteoporosis.

Long-term glucocorticoid treatment results in reduced mineral density. The effect of GCs on bone is dominated by its inhibitory effect on bone formation (Bultink et al., 2013). The GC-induced suppression of osteoblast differentiation is one of the mechanisms by which GCs reduce bone formation (Hsu and Nanes, 2017). The ERK signaling pathway has been intensively investigated in regulating osteoblast differentiation. Studies have shown that ERK is constantly activated during osteogenic differentiation, and ERK phosphorylates and activates Runx2, thereby promoting osteogenic differentiation (Jaiswal et al., 2000; Lai et al., 2001; Zhang et al., 2012). Thus, ERK signaling pathway plays a crucial role in the differentiation of osteoblasts.

Geniposide, derived from the dried fruit of *Gardenia jasminoides* Ellis, has been reported to have anti-oxidative (Li et al., 2018; Lu et al., 2018), anti-inflammatory (Deng et al., 2018; Wang et al., 2018), anti-viral (Zhang et al., 2017), anti-tumor (Ma and Ding, 2018), and neuroprotective effects (Chen et al., 2015). It has been found that geniposide ameliorates trinitrobenzene sulfonic acid (TNBS)-induced experimental rat colitis and histopathological changes of mesenteric lymph node in collagen-induced arthritis (CIA) rats (Wang et al., 2017; Xu et al., 2017). Studies also show that geniposide stimulates insulin secretion in pancreatic β-cells by regulating glucagon-like peptide-1 (GLP-1) receptor and promotes β-cell regeneration and survival (Yao et al., 2015; Zhang et al., 2016; Liu et al., 2017). In addition, studies have indicated that geniposide protects against cell injury in post-ischaemic neurovascular and Aβ-induced damage (Sun et al., 2014; Huang et al., 2017). However, the effects of geniposide in GC-induced osteogenic suppression remain unknown. Therefore, the present study investigated the effects and underlying mechanisms of geniposide on dexamethasone (DEX)-induced suppression of osteogenesis in MC3T3-E1 cells.

## Materials and Methods

### Reagents and Cell Culture

**FIGURE 1:**
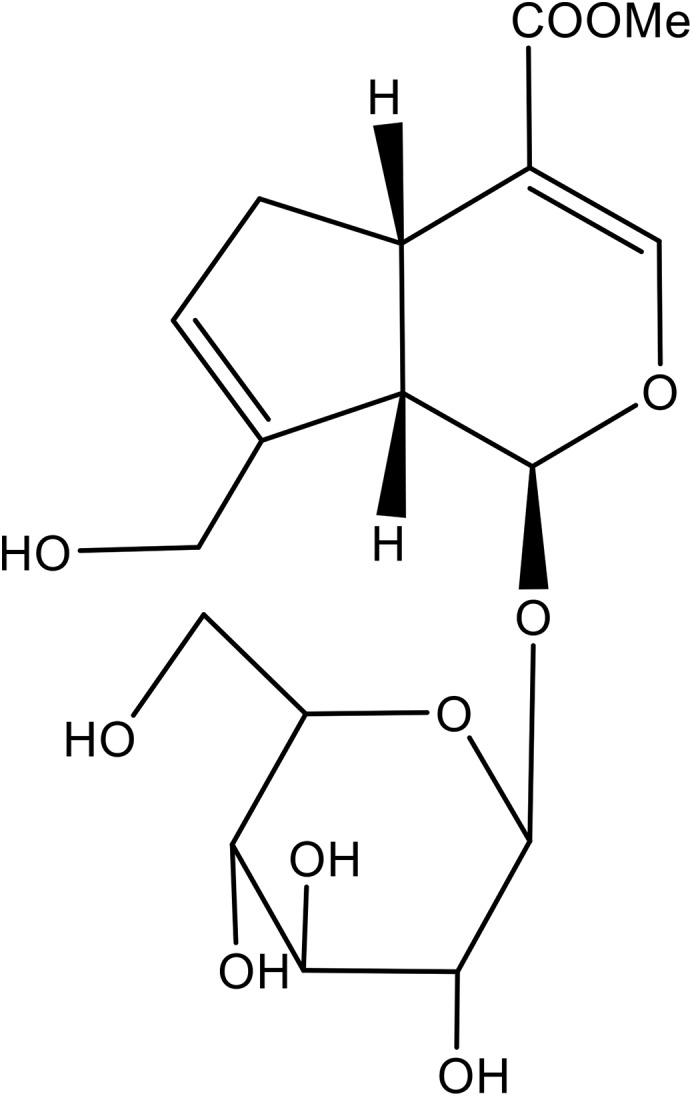
Chemical structure of geniposide.

Geniposide (Purity: >98%, Figure 1) was purchased from Chengdu Best Reagent Co., Ltd. (Chengdu, China). Dexamethasone (DEX), U0126 and exendin 9–39 were obtained from Sigma Chemical Co. (St. Louis, MO, United States). Cell Counting Kit-8 (CCK-8) was from Dojindo Laboratories (Japan). MC3T3-E1 cells were obtained from Chinese Academy of Sciences Cell Bank. Cells were grown in Modified Eagle’s Medium of Alpha (a-MEM) (Gibco) supplemented with 10% fetal bovine serum (FBS) (Gibco), 100 U/mL penicillin, and 100 μg/mL streptomycin (Gibco). For the induction of osteoblastic differentiation, MC3T3-E1 cells were incubated in osteogenic induction medium (OIM, α-MEM, 10% fetal bovine serum, 10 mM β-glycerophosphate, and 50 μg/mL ascorbic acid).

### Cell Viability Assay

Samples (5 × 10^3^ per well) were subcultured in a 96 flat-bottomed well plate. After 24 h, cells were treated with geniposide at different concentrations for 1, 2, 3, and 7 days. The cell viability was assessed by using the Cell Counting Kit-8 (CCK-8). The absorbance at 450 nm was measured with a microplate reader.

### Alkaline Phosphatase (ALP) Activity Assay

Cells were washed twice with phosphate buffer saline (PBS) and then lysed in 0.1% (v/v) Triton X-100 in PBS for 30 min. The lysates were centrifuged at 12,000 rpm for 10 min at 4°C, and the supernatants were harvested. The ALP activity was detected by using the ALP assay kit (Beyotime, China). The protein concentration of cell lysates was measured by using the bicinchoninic acid (BCA) protein assay. The ALP activity was normalized to the total protein concentration.

### ALP Staining

ALP staining was performed by using BCIP/NBT solution (Sigma). Briefly, the medium was removed, and the cells were rinsed twice with PBS. The cells were fixed with 70% ethanol for 10 min and equilibrated with ALP buffer (0.15 M NaCl, 0.15 M Tris–HCl, 1 mM MgCl_2_, pH 9.5) for 15 min. Then, the cells were incubated with NBT-BCIP solution (Sigma) at 37°C in dark for 30 min. The reaction was stopped by deionized water, and the plates were dried and taken photos.

### Mineralization Assay

Cells were washed twice with PBS and fixed with 70% ethanol for 10 min. Then, cells were incubated with a 0.5% Alizarin Red S (pH 4.1) for 10 min at room temperature. Orange red staining indicated the position and intensity of calcium deposits. To quantify the Alizarin Red S staining, 10% cetylpyridinium chloride (CPC, Sigma) was added to each well and incubated for 30 min. The optical density (OD) of the extract was measured at 550 nm.

### RNA Extraction and Real-Time PCR

Total RNA was extracted from the cultured cells with TRIZOL reagent, and the cDNAs were synthesized by using a Prime Script^>TM^ RT reagent Kit with gDNA eraser (TaKaRa, China). SYBR Premix Ex TaqII Reverse Transcriptase (TaKaRa, China) was used for quantitative real-time PCR (qRT-PCR), which was performed by using a 7500 real-time PCR system (Applied Bio-systems, United States). Amplification conditions were as follows: 95°C for 30 s, 40 cycles of 95°C for 5 s, and 60°C for 34 s. Sense and antisense primers of osteopontin (OPN), Runt-related transcription factor 2 (Runx2), and Osterix (Osx) were designed by primer 5.0 software and were shown in Table 1. The relative expression of mRNA was evaluated by the 2^-ΔΔCt^ method and normalized to the expression of β-actin.

**Table 1:** Primers used for quantitative real-time PCR.

Gene	Forward (5′–3′)	Reverse (5′–3′)
OPN	tccaaagccagcctggaac	tgacctcagaagatgaactc
Runx2	gaatgcactacccagccac	tggcaggtacgtgtggtag
Osx	aggaggcacaaagaagccatac	agggaagggtgggtagtcatt
β-actin	gccaaccgtgaaaagatgac	accagaggcatacagggacag

### Western Blotting Analysis

Cells were lysed by the protein extraction reagent (M-PER, Pierce, Illinois) plus the protease inhibitor cocktail (Halt, Pierce) for 30 min on ice. Protein concentrations were determined by using the BCA assay (Beyotime, China). Equal proteins were fractionated by 10% SDS-polyacrylamide gel, and then proteins were transferred to polyvinylidene difluoride (PVDF) membrane (Whatman, United States). The membrane was blocked with 2% bovine serum albumin (BSA) in Tris-buffered saline-Tween 20 (0.1%) (TBS-T) for 1 h at room temperature. After that, the membrane was incubated with anti-Runx2 (Abcam, ab23981, 1:1000), anti-Osx (Abcam, ab22552, 1:1000), anti-OPN (Abcam, ab8448, 1:1000), anti-ERK1/2 (Cell Signaling Technology, #4695, 1:1000), anti-p-ERK1/2 (Cell Signaling Technology, #4370, 1:1000), or anti-β-actin antibodies (Beyotime, AF0003, 1:1000) overnight at 4°C. The membrane was washed, and the bound primary antibodies were detected by incubating for 2 h with horseradish peroxidase-conjugated goat anti-rabbit (Abcam, ab6721, 1:5000) or anti-mouse (Abcam, ab6789, 1:5000) secondary antibody. Finally, the membrane was washed three times with TBST and developed with enhanced chemiluminescence (ECL) kit (GE Healthcare, Beijing, China). The relative quantities of proteins were determined by scanning densitometry (ChemiDoc XRS1 Systems Bio-Rad, Hercules, United States).

### Statistical Analysis

All data were presented as mean ± SD, and statistical analysis was performed by using one-way analysis of variance. A value of *p* < 0.05 was considered statistically significant.

## Results

### Effect of Geniposide on Viability of MC3T3-E1 Cells

**FIGURE 2:**
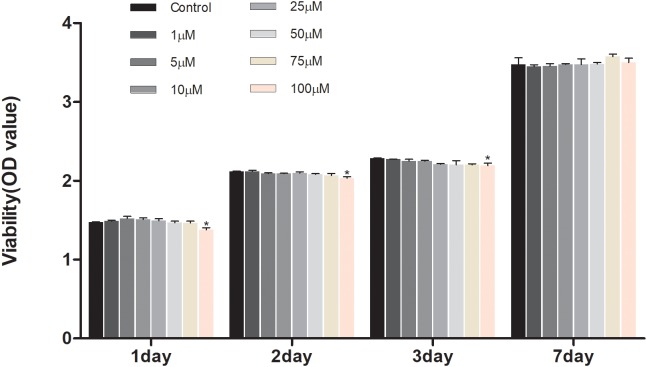
Effects of geniposide on viability of MC3T3-E1 cells. The cells were treated with geniposide at different concentrations for 1, 2, 3, and 7 days, and the cell viability was performed by using CCK-8 assay. *n* = 3. ^∗^*p* < 0.05 compared with Control.

To investigate the effect of geniposide on cell viability, the CCK-8 assay was performed. The result showed that geniposide did not influence the cell viability at the doses range from 1 to 75 μM on days 1, 2, 3, and 7 (Figure 2).

### Geniposide Alleviated the Inhibitory Effect of DEX on Alkaline Phosphatase (ALP) Activity

**FIGURE 3:**
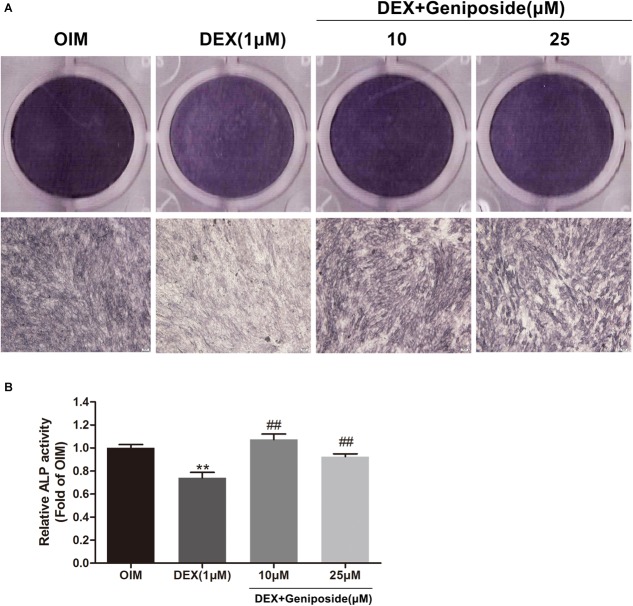
Geniposide alleviated the inhibitory effect of DEX on ALP in MC3T3-E1 cells. **(A)** ALP staining was performed with BCIP/NBT kit after the MC3T3-E1 cells were treated with different dosages of geniposide and DEX for 5 days. **(B)** The ALP activity was measured after incubation of cells with different dosages of geniposide and DEX for 5 days. OIM, osteogenic induction medium; DEX, dexamethasone. *n* = 3. ^∗∗^*p* < 0.01 compared with OIM; ^##^*p* < 0.01 compared with DEX.

ALP is an early marker of osteogenic differentiation, so we studied the effect of geniposide on the ALP activity in DEX-treated MC3T3-E1 cells. We found that DEX inhibited the ALP activity in MC3T3-E1 cells. In contrast, geniposide significantly alleviated the inhibitory effect of DEX on ALP activity (*p* < 0.01) (Figure 3). In addition, similar results were observed by ALP staining (Figure 3).

### Geniposide Attenuated the Inhibitory Effect of DEX on Mineralization

**FIGURE 4:**
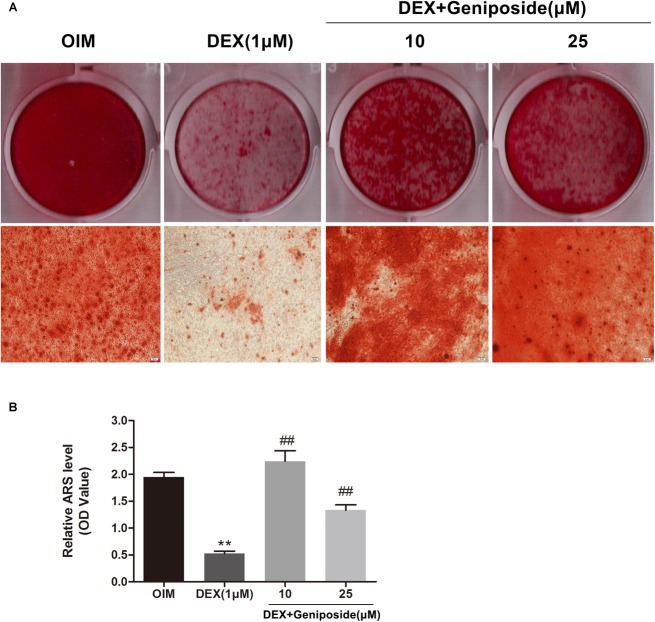
Geniposide attenuated the inhibitory effect of DEX on mineralization. **(A)** The mineralized nodules were stained by Alizarin Red S after treatment of MC3T3-E1 cells with DEX and geniposide in OIM for 14 days. **(B)** The mineralization was quantified by extraction of Alizarin Red S dye with 10% CPC. OIM, osteogenic induction medium; DEX, dexamethasone. *n* = 3. ^∗∗^*p* < 0.01 compared with OIM; ^##^*p* < 0.01 compared with DEX.

In addition, we examined the effects of DEX and geniposide on the mineralization of MC3T3-E1 cells. We found that DEX inhibited the calcium nodule formation compared with OIM. Geniposide increased calcium deposition compared with DEX (Figure 4).

### Geniposide Increased Expression of OPN, Runx2, and Osx mRNA in DEX-Treated MC3T3-E1 Cells

**FIGURE 5:**
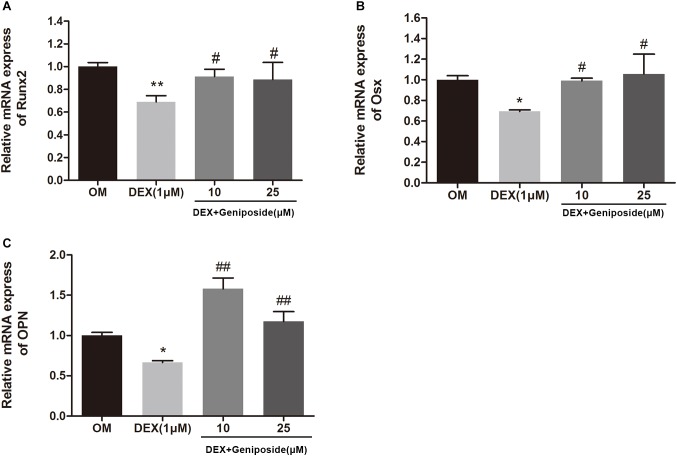
Geniposide increased the expression of OPN, Runx2, and Osx mRNA in MC3T3-E1 cells. MC3T3-E1 cells were treated with DEX and geniposide in OIM for 3 days, and then the gene expression of Runx2 **(A)**, Osx **(B)**, and OPN **(C)** was detected by qRT-PCR. OIM: osteogenic induction medium. DEX, dexamethasone. *n* = 3. ^∗^*p* < 0.05, ^∗∗^*p* < 0.01 compared with OIM; ^#^*p* < 0.05, ^##^*p* < 0.01 compared with DEX.

To confirm the effect of geniposide on osteogenesis in MC3T3-E1 cells, the mRNA expression of key marker genes OPN, Runx2, and Osx was assessed. The results showed that DEX significantly downregulated the expression of OPN, Runx2, and Osx (Figure 5). However, geniposide significantly increased the expression of OPN, Runx2, and Osx compared with DEX (Figure 5).

### Geniposide Increased OPN, Runx2, and Osx Protein Expression in DEX-Treated MC3T3-E1 Cells

**FIGURE 6:**
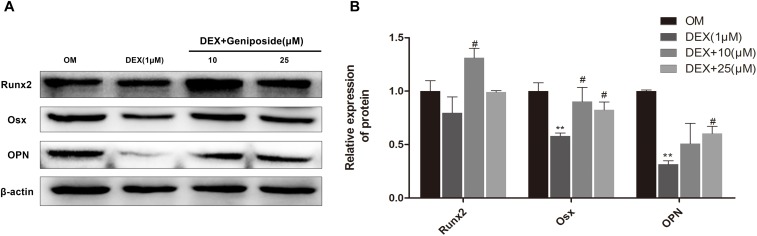
Geniposide increased OPN, Runx2, and Osx protein expression in MC3T3-E1 cells. **(A)** The total proteins were separated by 10% SDS-PAGE and detected with the indicated antibodies of OPN, Runx2, and Osx after MC3T3-E1 cells were treated with DEX and geniposide in OIM for 3 days. β-actin was used as the loading control. **(B)** The bar charts showed the quantification of OPN, Runx2, and Osx. OIM: osteogenic induction medium. DEX, dexamethasone. *n* = 3. ^∗∗^*p* < 0.01 compared with OIM; ^#^*p* < 0.05 compared with DEX.

We further studied the effect of geniposide on the protein expression of OPN, Runx2, and Osx in MC3T3-E1 cells. The results showed that the protein expression of OPN, Runx2, and Osx was significantly decreased in DEX group, and geniposide significantly increased the protein expression of OPN, Runx2, and Osx compared with DEX (Figure 6).

### Geniposide Activated ERK Signaling Pathway in DEX-Treated MC3T3-E1 Cells

**FIGURE 7:**
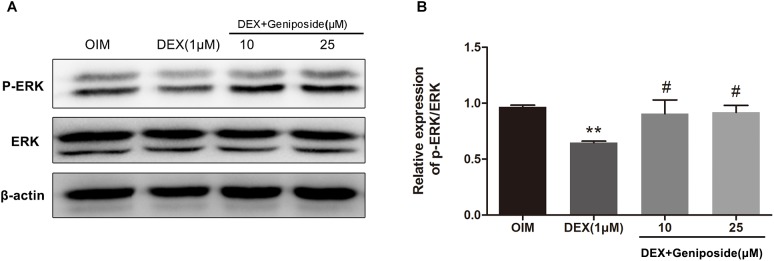
Geniposide activated ERK pathway in DEX-treated MC3T3-E1 cells. **(A)** The expression of p-ERK was detected by western blotting after MC3T3-E1 cells were treated with DEX and geniposide in OIM for 1 day. β-actin was used as an internal reference. **(B)** The bar charts showed the quantification of the ratio of p-ERK/ERK. OIM, osteogenic induction medium; DEX, dexamethasone. *n* = 3. ^∗∗^*p* < 0.01 compared with OIM; ^#^*p* < 0.05 compared with DEX.

It has been shown that ERK pathway is important for osteogenic differentiation. We determined the levels of ERK and phosphorylated ERK (p-ERK) in DEX-treated MC3T3-E1 cells by using western blot assay. Western blot analysis showed that DEX inhibited ERK phosphorylation, and geniposide restored the level of p-ERK (Figure 7).

### U0126 Inhibited the Protective Effect of Geniposide in DEX-Treated MC3T3-E1 Cells

**FIGURE 8:**
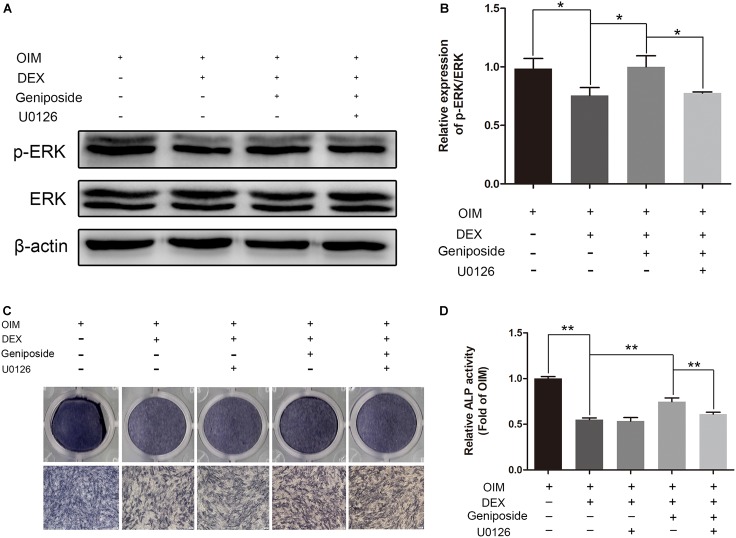
U0126 inhibited the protective effect of geniposide. **(A)** The expression of p-ERK was detected by western blotting after the MC3T3-E1 cells were pretreated with 10 μM U0126 for 1 h, and followed by DEX and geniposide treatment for 1 day. β-actin was used as an internal reference. **(B)** The bar charts showed the quantification of the ratio of p-ERK/ERK. **(C)** ALP staining was performed with BCIP/NBT kit after the MC3T3-E1 cells were pretreated with 10 μM U0126 for 1 h, and followed by DEX and geniposide treatment for 5 days. **(D)** The ALP activity was measured after the MC3T3-E1cells were pretreated with 10 μM U0126 for 1 h, and followed by DEX and geniposide treatment for 5 days. OIM, osteogenic induction medium; DEX, dexamethasone. *n* = 3. ^∗^*p* < 0.05, ^∗∗^*p* < 0.01.

To further elucidate the role of ERK in the protective effect of geniposide, cells were pretreated with 10 μM U0126 (an inhibitor of ERK activation), and followed by DEX and geniposide treatment. As shown in Figure 8, U0126 abrogated geniposide-induced phosphorylation of ERK, suggesting that U0126 abolished the geniposide-induced activation of ERK. In addition, U0126 inhibited the protective effect of geniposide (Figure 8).

### Exendin 9-39 Inhibited the Protective Effect of Geniposide in DEX-Treated MC3T3-E1 Cells

**FIGURE 9:**
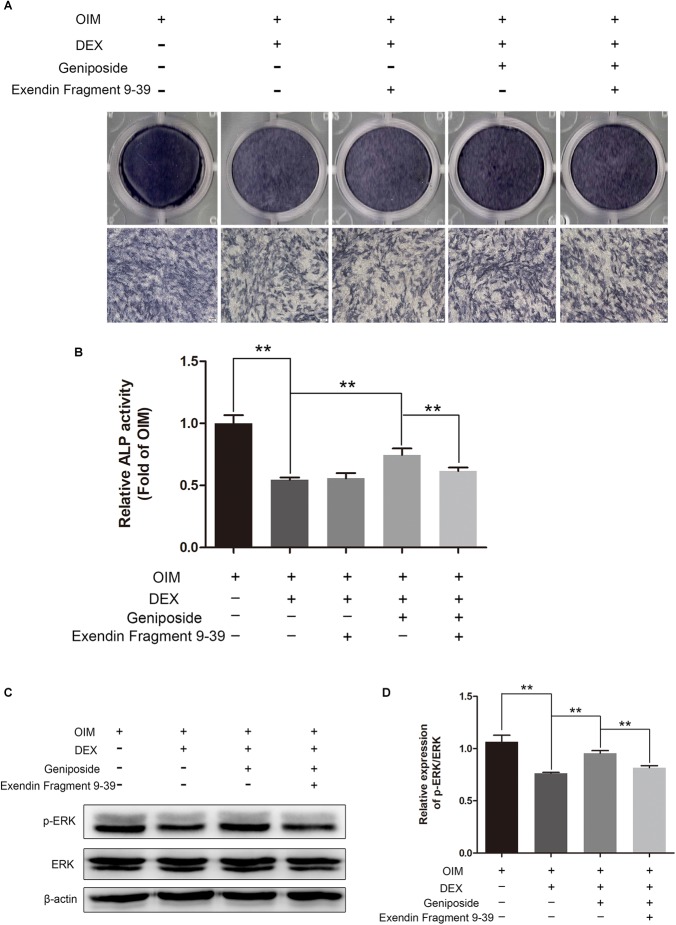
Exendin 9–39 inhibited the protective effect of geniposide in DEX-Treated MC3T3-E1 cells. **(A)** ALP staining was performed with BCIP/NBT kit after the MC3T3-E1 cells were pretreated with 200 nM exendin 9–39 for 1 h, and followed by DEX and geniposide treatment for 5 days. **(B)** The ALP activity was measured after the cells were pretreated with 200 nM exendin 9–39 for 1 h, and followed by DEX and geniposide treatment for 5 days. **(C)** The expression of p-ERK was detected by western blotting after the MC3T3-E1 cells were pretreated with 200 nM exendin 9–39 for 1 h, and followed by DEX and geniposide treatment for 1 day. β-actin was used as an internal reference. **(D)** The bar charts showed the quantification of the ratio of p-ERK/ERK. OIM, osteogenic induction medium; DEX, dexamethasone. *n* = 3. ^∗∗^*p* < 0.01.

To investigate further the mechanism of geniposide, we determined the effect of GLP-1 receptor antagonist exendin 9–39 on protective effect of geniposide on GC-induced suppression of osteogenic differentiation. We found geniposide increased the ALP activity in DEX-treated MC3T3-E1 cells and exendin 9–39 inhibited the effect of geniposide. Furthermore, exendin 9–39 abolished the geniposide-induced activation of ERK (Figure 9).

## Discussion

In the present study, for the first time, we studied the effects of geniposide on DEX-induced osteogenic suppression. First, we investigated the effects of geniposide on viability of MC3T3-E1 cells. We found that different doses of geniposide ranging from 1 to 75 μM did not affect the viability of MC3T3-E1 cells, indicating that geniposide was not cytotoxic to MC3T3-E1 cells in a wide range of concentrations.

Next, we evaluated the effect of geniposide on the ALP activity in DEX-treated MC3T3-E1 cells. Our results indicated that DEX inhibited the activity of ALP in MC3T3-E1 cells. In contrast, geniposide significantly increased the activity of ALP in DEX-treated MC3T3-E1 cells. In addition, we found that DEX inhibited the calcified nodule formation in MC3T3-E1 cells. Geniposide significantly promoted the formation of calcified nodule in DEX-treated MC3T3-E1 cells. These results showed that geniposide alleviated the suppressive effects of DEX on osteogenic differentiation in MC3T3-E1 cells.

Runx2 is a major transcription factor and essential for osteoblast differentiation (Ducy et al., 1997; Komori et al., 1997). Osx is another transcription factor and plays a major role in bone formation (Nakashima et al., 2002; Kim et al., 2006). Osx acts as a downstream factor of Runx2 (Miraoui et al., 2009). OPN is a prominent bone matrix protein and a typical marker of osteoblast (Yao et al., 1994; Tu et al., 2006). Both Runx2 and Osx bind to the promoter of OPN and upregulate its expression (Ducy et al., 1997). In this study, we found that DEX downregulated the expression levels of OPN, Runx2, and Osx. However, geniposide significantly upregulated the expression levels of OPN, Runx2, and Osx in Dex-treated MC3T3-E1 cells. The results suggested that geniposide attenuated the suppressive effects of DEX through mediating transcription factors including Runx2 and Osx.

Recent reports have shown that GLP-1 plays a vital role in bone formation, and GLP-1 receptor agonist increases osteoblast activity (Meng et al., 2016; Wu et al., 2017). The GLP-1 receptor is expressed in MC3T3-E1 cells (Aoyama et al., 2014; Wu et al., 2017). Studies showed that geniposide was a GLP-1 receptor agonist (Gong et al., 2014; Zhang et al., 2016). Our results showed that geniposide attenuated inhibitory effect of osteogenic differentiation induced by DEX, and the effect of geniposide against inhibitory effect of osteogenic differentiation was decreased with GLP-1 receptor antagonist exendin 9–39, suggesting that GLP-1 receptor was involved in the protection of geniposide against inhibitory effect of osteogenic differentiation.

Studies have shown that ERK signaling pathway plays a crucial role in the differentiation of osteoblasts (Jaiswal et al., 2000; Lai et al., 2001). GCs are known to regulate the activation of ERK (Poulsen et al., 2011; Frenkel et al., 2015). We also found that GCs inhibited the activity of ERK. In addition, Kou et al. showed that geniposide increased the expression and phosphorylation of ERK in primary hepatocytes (Kuo et al., 2005). A study by Huang et al. showed that geniposide activated ERK pathway in a rat model of experimental stroke (Huang et al., 2017). However, studies showed that geniposide inhibited ERK signaling pathway in ischemia/reperfusion-induced renal injury and oxygen/glucose deprivation-induced brain microvascular endothelial cells (Li et al., 2016; Ye et al., 2016). These studies suggest that geniposide has different effects on ERK pathway in different cell types. Thus, we explored the roles of geniposide on ERK pathway in DEX-treated MC3T3-E1 cells. We found that geniposide significantly increased the ERK phosphorylation. The ERK activation inhibitor and GLP-1 receptor antagonist abolished the geniposide-induced activation of ERK and inhibited the protective effect of geniposide. These data indicated that ERK pathway was involved in the biological effects of geniposide. Furthermore, ERK mediates Runx2 phosphorylation and the transcriptional activity in bone (Ge et al., 2007, 2009). Thus, the effects of geniposide on Runx2 and Osx expression might be mediated by activating ERK signaling pathway via GLP-1 receptor.

## Conclusion

In summary, we demonstrated that geniposide alleviated GC-induced osteogenic suppression in MC3T3-E1 cells. The effects of geniposide were at least partially associated with activating ERK signaling pathway via GLP-1 receptor. Geniposide might be a potential therapeutic agent for protection against GC-induced osteoporosis.

## Author Contributions

BX, DX, CZ, and LW participated in research design. BX, JW, YL, XW, and ZZ conducted the experiments. BX, DX, and LW contributed new reagents or analytic tools and wrote or contributed to the writing of the manuscript. BX, JW, CZ, and DX performed the data analysis.

## Conflict of Interest Statement

The authors declare that the research was conducted in the absence of any commercial or financial relationships that could be construed as a potential conflict of interest.
